# Clinical study of the factors affecting radioulnar deviation of the wrist joint

**DOI:** 10.1186/1471-2474-11-9

**Published:** 2010-01-15

**Authors:** Panagiotis Kitsoulis, Georgios Paraskevas, Kalliopi Iliou, Panagiotis Kanavaros, Aikaterini Marini

**Affiliations:** 1 Medical School, University of Ioannina, Panepistimiou Avenue, Ioannina, Greece; 2 Medical School, University of Thessaloniki, Panepistimiou Avenue, Thessaloniki, Greece

## Abstract

**Background:**

The radioulnar carpal joint is critical for hand and wrist function. Radioulnar deviation indicates distal radioulnar joint flexibility and reflects the structure and function of the carpal bones, ulna, radius and ligaments. The present study examined whether radioulnar deviation is affected by gender, manual labor, playing a musical instrument, playing sport, handedness, previous fracture or prior inflammation. The study used clinical findings based on anatomical landmarks

**Methods:**

The ulnar, radial and total deviations for both left and right hands were measured in 300 subjects (157 men and 143 women) of mean age 21.7 years. Measurements were made with the forearm in a fixed pronated position using a novel specially designed goniometer. The gender of each subject was recorded, and information on playing of sport, playing a musical instrument, manual labor, handedness, and history of fracture or inflammation was sought. Data were analyzed using a multifactor ANOVA test.

**Results:**

No statistically significant difference (p-value > 0.05) was found between those comparing groups except the total deviation of athletes' left hand versus the total deviation of non athletes' left hand (p-value 0.041 < 0.05) and the radial deviation of manual workers' left hand and non manual workers' left hand (p-value 0.002 < 0.05).

**Conclusions:**

This study was based on clinical findings using anatomical landmarks. We found that manual workers and athletes showed greater left hand flexibility. This suggests that activities that place chronic stress on the radiocarpal joint can independently affect radioulnar deviation.

## Background

The distal radioulnar joint is critical in normal hand and wrist function. Radioulnar deviation describes hand movements towards the ulna and the radius, and characterizes the entire radioulnar carpal joint [1]. Wrist joint flexibility is controlled by several bones and ligaments, namely, the radial and ulnar collateral ligaments, the discus articularis, the intercarpal ligaments, and the volar and dorsal radiocarpal ligaments [2]. The head of the ulnar, the ulnar notch of the radius, the distal end of the radius, and the lunate and scaphoid bones are also involved, whereas the styloid process of the ulna and radius are responsible for impedance of lateral and medial wrist movements [3].

Wrist joint movements are also determined by the shape and contour of contact surfaces and surrounding soft tissues [4]. Distal radioulnar joint movements include pronation and supination in collaboration with the proximal radioulnar joint. A middle radioulnar union is formed by the oblique cord and the interosseous membrane which also affect the range of motion. Movement at the distal radioulnar joint is also determined by the relative movement of the radius, ulna, carpal bones, ligaments and anatomical variations of the wrist joint [5].

The articular surfaces of the ulna, radius and carpal bones are engaged during many activities, such as sport, manual work and musical instrument playing. These surfaces are also affected by fractures. The range of radioulnar deviation indicates the flexibility of the radioulnar carpal joint.

Several authors have measured radioulnar deviation using radiological techniques. The present study sought to identify factors affecting radioulnar deviation. We determined the effect of gender, playing of sport, playing a musical instrument, manual labor, handedness, previous fracture, and prior inflammation on ulnar, radial, and total deviations in both the right and left hands of 300 volunteers at the University of Ioannina. It is the first time that clinical measurement of radioulnar deviation was performed using an enlarged goniometer. The hypothesis is that no statistically significant difference exists between the comparing groups.

## Methods

### Material

The study was performed in the Laboratory of Anatomy at the Medical School of the University of Ioannina. The project involved 300 volunteers composed of 157 men and 143 women aged from 18 to 24 years (mean age 21.7 years). All volunteers were healthy adults with normally developed musculoskeletal systems.

**Figure 1:**
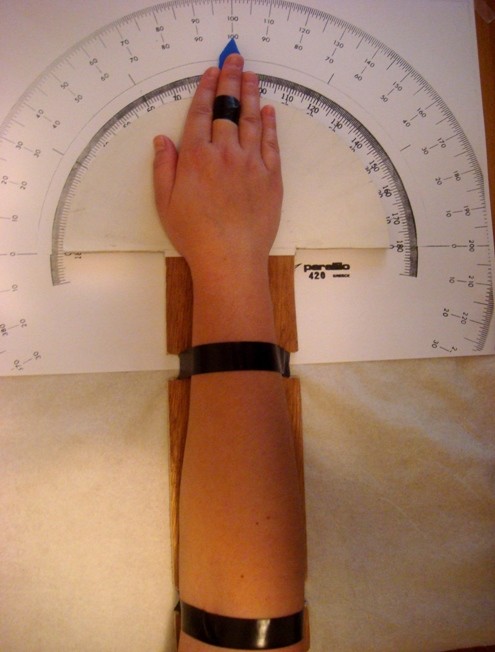
**Right hand in the neutral position**.

Maximum radioulnar deviation was measured using a specially designed novel goniometer. The goniometer was printed on a table (30 × 45 cm) and placed on an examination desk. The forearm was fixed in pronation onto a stiff surface to a accurately and specifically determine the flexibility of the distal radioulnar joint. The forearm was held totally immobile using two straps firmly bolted to the desk. The center of motion was determined as the point where an imaginary axis passing through the middle finger and third metacarpal bone met the halfway point of the distance between the styloid processes of the ulna and radius, as described by Kapandji [6]. This center of rotation was placed over the zero point of the goniometer. A plastic marker was stabilized with a bandage under the middle finger. A line parallel to the long axis of the forearm defined the place where the middle finger and the marker should be placed (Fig. 1). This was termed the neutral position, and was the position from which every movement began. All fingers remained close together with the palmar surface facing the goniometer throughout testing. Hands were not permitted to lift during testing, thus the measured angle was the result of movement of the distal radioulnar joint only in the horizontal plane.

**Figure 2:**
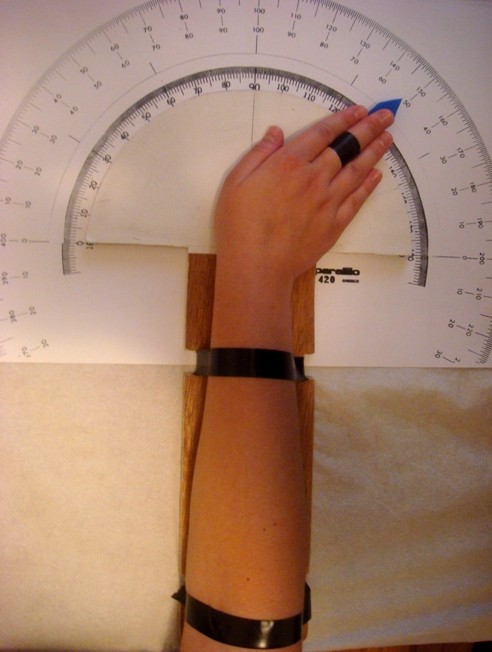
**Right hand ulnar deviation**.

From the neutral position, each subject was asked to move the hand as far as possible toward the ulna, and a measurement was taken. Subjects were next asked to move the hand from the neutral position, as far as possible toward the radius and another measurement was recorded. The radial and ulnar angles were noted with the neutral position given the value of 90 degrees. Thus, an ulnar movement of 130 degrees indicated an angle of movement of 40 degrees (*i.e. *130-90 degrees) which represented the ulnar deviation (Fig. 2). Only the maximum active (not passive) deviation was measured. The marker indicating the degrees of deviation was flat and located between the middle finger and the goniometer; the marker did not obscure movements. Measurements were taken for both for both the right and left hand. At the completion of testing the gender of each subject was recorded, and we determined whether a subject played sport, played a musical instrument, was a manual labourer, which was the dominant hand (left *vs. *right *vs. *ambidextrous) and whether any fracture or inflammation (*i.e. *tendonitis) involving the wrist, fingers, ulna or radius had ever been experienced. An athlete was characterized as a volunteer who had played sports involving the wrist joint for more than 5 years of his life, while musicians were defined as those who had played a musical instrument for more than 8 years. A person was defined as ambidextrous if deliberately or naturally used both hands for writing, drawing, playing an instrument, or during sport. A manual worker was characterized as a volunteer who had done physical work involving his hands for more than 5 years of his life.

No volunteer had rheumatoid arthritis. Each subject was measured and assessed twice under the supervision of two observers.

All subjects gave written informed consent to participate in this study and to publish the data acquired. The research was approved by the Ethics Committee of the Medical School of University of Ioannina.

### Statistical analysis

Our variables were the radial, the ulnar and total deviation of left and right hand. The comparing groups were women-men, athletes-non athletes, musicians-non musicians, previous fracture-non previous fracture, previous inflammation-non previous inflammation, left handed-right handed, manual workers-non manual workers. There were 255 right-handed and 45 left-handed subjects. There were 139 musicians, 83 athletes and 38 manual workers. Previous fractures were recorded in 48 subjects and all involved breaks in the right hand. Previous inflammation had occurred in 53 subjects, 20 in the left and 33 in the right hand.

Analysis was performed on anonymous data using SPSS version 13.0. Kolmogorov-Smirrnov and Sharpiko-Wilk tests of normality were employed to determine whether data were normally distributed. Categorical and continuous variables were compared using a multifactor ANOVA test. A p-value of less than 0.05 was considered to represent a statistically significant difference. The confidence interval was 95%.

## Results

**Figure 3:**
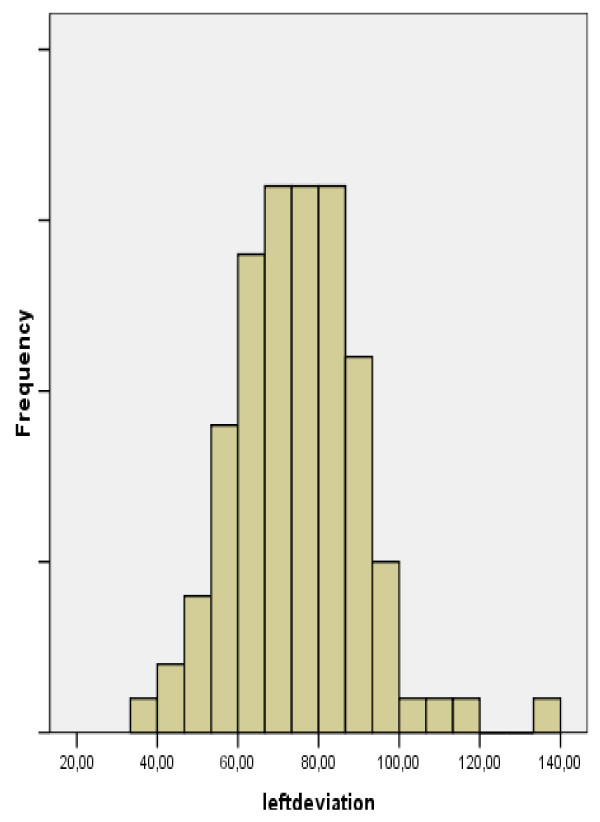
**Graph depicting radioulnar total deviation in the left hand of athletes and non-athletes**.

**Figure 4:**
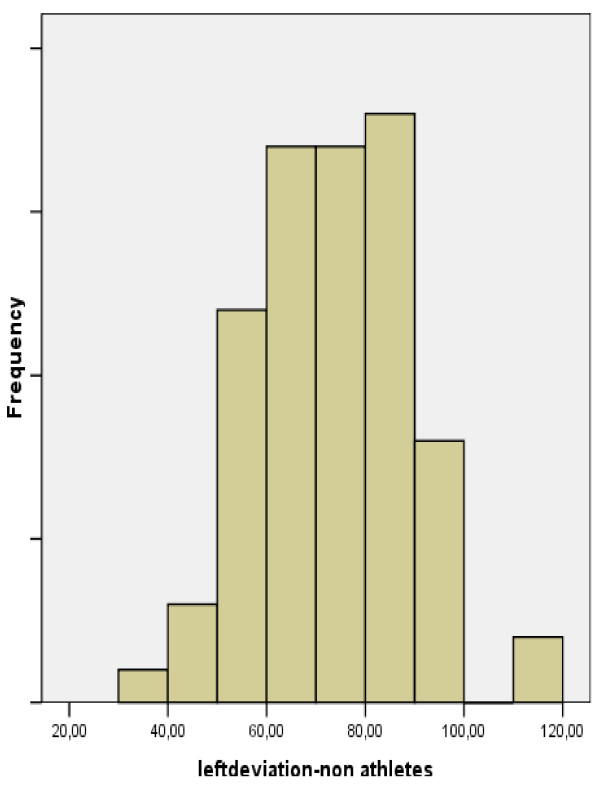
**Graph depicting total deviation in the left hand of non-athletes**.

**Figure 5:**
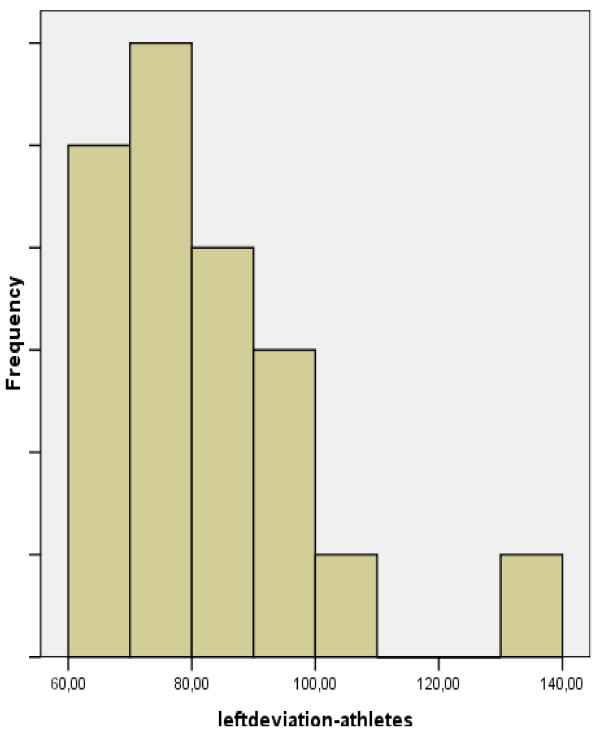
**Graph depicting total deviation in the left hand of athletes**.

The ulnar, radial, ad total deviations of the left and right hand of each subject were measured. Subjects were classified by gender, handedness, and whether they were athletes, manual workers, played a musical instrument, or had experienced previous fracture or inflammation. The hypothesis of our study was the equality of our variables' means between our comparing groups. We recorded data in tables and performed a multifactor ANOVA test. We performed a multifactor ANOVA test and we found statistically significant difference in the total deviation of left hand between athletes and non-athletes (p = 0.041 < 0.05) [Tables 1, 2, 3, 4] (Fig. 3, 4, 5). We also found statistically significant difference for the radial deviation of left hand between manual and non-manual workers (p = 0.002 < 0.05) [Tables 5, 6, 7, 8]. There was no statistically significant difference in the total deviation of right hand between athletes and non-athletes [Tables 9, 10]. We also did not find statistically significant difference between manual and non-manual workers for the radial deviation of right hand [Tables 11, 12]. We found no statistically significant difference for our variables in the other comparing groups [Tables 13, 14].

**Table 1:** Descriptive statistics for total deviation of the left hand.

	N	Range	Minimum	Maximum	Mean	Std. Deviation
**Total Deviation** **(left hand)**	300	97.0	38.0	135.0	74.7	15.8

**Table 2:** Group statistics for left hand total deviation in athletes and non-athletes.

	Athlete	N	Mean	Std. Deviation	Std. Error Mean
**Total Deviation (left hand)**	**yes**	83	81.6	17.0	3.8
	**no**	217	73.0	15.4	1.7

**Table 3:** Test of normality for total deviation of left hand. There is a normal distribution.

	Kolmogorov-Smirnov			Shapiro-Wilk		
	
	Statistic	df	Sig.	Statistic	df	Sig.
**Total Deviation** **(left hand)**	.057	98	.200	.97	98	.089

**Table 4:** Multifactor ANOVA test. Dependent Variable: Total deviation of left hand

Source	Sig.
Gender	.677

Musicians	.558

Previous fracture	.540

Athletes	.041

Previous inflammation	.181

Handedness	.595

Manual labourer	.052

**Table 5:** Descriptive statistics for radial deviation of the left hand.

	N	Range	Minimum	Maximum	Mean	Std Deviation
**Radial Deviation** **(left hand)**	300	53.0	11.0	64.0	29.0	8.3

**Table 6:** Group statistics for left hand radial deviation in manual and non-manual workers.

	manual worker	N	Mean	Std Deviation	Std Error Mean
**Radial Deviation (left hand)**	**yes**	38	41.0	20.2	11.7
	**no**	262	28.5	7.6	.77

**Table 7:** Test of normality for radial deviation measurements of the left hand.

	Kolmogorov-Smivov			Shapiro-Wilk		
	
	Statistic	df	Sig.	Statistic	df	Sig.
**Radial Deviation** **(left hand)**	.09	98	.05	.95	98	.003

**Table 8:** Multifactor ANOVA Test. Dependent Variable: Radial deviation of left hand

Source	Sig.
Gender	.493

Musicians	.734

Previous fracture	.873

Athletes	.508

Previous inflammation	.970

Handedness	.861

Manual labourer	.002

**Table 9:** Group statistics for right hand total deviation in athletes and non-athletes.

	Athlete	N	Mean	Std Deviation	Std Error Mean
**Total Deviation** **(right hand)**	**yes**	83	80.0	14.9	3.3
	**no**	217	73.3	14.4	1.6

**Table 10:** Multifactor ANOVA Test. Dependent Variable: Total deviation of right hand

Source	Sig.
Gender	.794

Musicians	.643

Previous fracture	.171

Athletes	.704

Previous inflammation	.060

Handedness	.171

Manual labourer	.178

**Table 11:** Group statistics for right hand radial deviation in manual and non-manual workers.

	Manual worker	N	Mean	Std. Deviation	Std. Error Mean
**Radial Deviation (right hand)**	**yes**	38	38.0	8.9	5.1
	**no**	262	27.5	7.8	0.8

**Table 12:** Multifactor ANOVA Test. Dependent Variable: Radial deviation of right hand

Source	Sig.
Gender	.915

Musicians	.420

Previous fracture	.072

Athletes	.968

Previous inflammation	.374

Handedness	.884

Manual labourer	.200

**Table 13:** Multifactor ANOVA Test. Dependent Variable: Ulnar deviation of left hand

Source	Sig.
Gender	.964

Musicians	.589

Previous fracture	.339

Athletes	.377

Previous inflammation	.065

Handedness	.391

Manual labourer	.535

**Table 14:** Multifactor ANOVA Test. Dependent Variable: Ulnar deviation of right hand

Source	Sig.
Gender	.421

Musicians	.992

Previous fracture	.677

Athletes	.623

Previous inflammation	.060

Handedness	.242

Manual labourer	.425

The mean and standard deviation for left and right hand of athletes, musicians, men, women, manual workers, right-handed, left-handed, history of inflammation or fracture are concentrated in Additional file 1.

## Discussion

The present study sought to identify factors affecting radioulnar deviation. We found that playing sport increased left hand total deviation (p-value 0.041 < 0.05), whereas manual labour increased left hand radial deviation (p-value 0.002 < 0.05). No statistically significant difference (p-value > 0.05) was found between the other comparing groups. Thus radioulnar deviation was not affected by gender, playing a musical instrument, previous fracture, prior inflammation, or handedness.

Unlike previous studies, the current work used clinical rather than radiological findings to determine radioulnar deviation. In addition, this is the first occasion on which our novel goniometer had been used to measure radioulnar deviation.

External landmarks such as the middle finger and the styloid process of the ulna and the radius were employed to determine the center of rotation. Distal and proximal radioulnar articulations and relevant ligaments affect the range of motion in the distal radioulnar joint. Therefore, we made measurements with the forearm in pronation as recommended elsewhere [7].

No statistically significant difference in the radioulnar deviation between men and women was found. This differs from a previous study that estimated radioulnar deviation using an electrogoniometer in combination with manual methods [8], where it was found that women had greater flexibility because of anatomical differences. Differing conclusions have also been drawn from elbow joint studies, with some fnding that women had greater flexibility [9] whereas others observed no difference between genders [1]. The lack of agreement between various studies exploring gender differences may reflect the use of distinct methodologies or the limitations in accuracy of our measurement method.

The present study found that a previous fracture or inflammation in the wrist or the distal radius or ulna did not affect radioulnar deviation. These findings appear to contradict those of previous studies showing that a history of inflammation or fractures reduced radioulnar deviation [1,10]. Kazuki and colleagues found that after a Colles fracture the mean radial and ulnar deviations each decreased by approximately 10 degrees [11]. Furthermore misalignment of a distal radial fracture obstructed distal radioulnar motion [12]. The range of motion can be affected by 20-25 degrees depending upon the amount of misalignment [13,14]. A previous arthrodesis attributable to inflammation aggravated movement of the distal and proximal rows of wrist bones [15] and thus limited the range of radioulnar deviation. The differences between the findings of previous studies and the current work may be attributable to the fact that the present study employed a younger population in whom the effect of injury was probably less severe.

We found that the radial deviation of manual workers' left hand presented a statistically significant difference in comparison to non-manual workers which implies greater flexibility for manual workers. Repetitive lifting activities affect the distal radius volar tilt angle and specifically the greater the forces are, the more the angle decreases [8,16]. Additionally, manual work can change the architecture of the carpal and ulnar bones, and consequently affect movement [17,18].

The mean radial deviation in the present study was 28.5 degrees, whereas the mean ulnar deviation was 45 degrees. These means differ from previously reported values of 15 and 30 degrees respectively [7]. The higher means of the present study probably reflect the use of a younger population.

## Conclusion

The present study found that flexibility of the radioulnar joint was not affected by gender, playing a musical instrument, handedness, or a history of fracture or inflammation. Athletes and manual workers present a more flexible radioulnar joint in their left hand regardless their sex and the aforementioned parameters. Those two groups present a statistically significant difference in radioulnar deviation of their left hand compared to non-athletes and non-manual workers. It is probable that these intense activities affect the carpal joint of the left hand in a person in such a grade that the vast of radioulnar deviation surpasses that of the other comparing groups. Furthermore, the fact that manual workers and athletes use both of their hands during their activities and the relevant forces are posed upon both of their hands could play a role in the flexibility of their hands.

## Competing interests

The authors declare that they have no competing interests.

## Authors' contributions

PK participated in design of the study, acquisition and interpretation of data and results and in drafting of the manuscript. He also gave the final approval for manuscript submission. GP and AM participated in design of the study, acquisition and interpretation of data and results, and in the drafting and revision of the manuscript. IK and PK performed statistical analyses and participated in interpretation of the data. All authors have read and approved of the final version of the manuscript.

## Pre-publication history

The pre-publication history for this paper can be accessed here:

http://www.biomedcentral.com/1471-2474/11/9/prepub

## Supplementary Material

Additional file 1 **Mean and standard deviation of the comparing groups**. Mean and standard deviation for left and right hand of athletes, musicians, men, women, manual workers, right-handed, left-handed, history of inflammation or fracture are concentrated here. Click here for file
