# Supplementary material for: Clinical study of the factors affecting radioulnar deviation of the wrist joint
Source: BMC Musculoskelet Disord. 2010 Jan 15;11:9. doi: 10.1186/1471-2474-11-9 (PMC2836282; doi:10.1186/1471-2474-11-9)
Supplement: Additional file 1 — Mean and standard deviation of the comparing groups. Mean and standard deviation for left and right hand of athletes, musicians, men, women, manual workers, right-handed, left-handed, history of inflammation or fracture are concentrated here. [file 1471-2474-11-9-S1.DOC]

|  | Mean | St Deviation |
| --- | --- | --- |
| Radial deviation of left hand | 31.8 | 9.6 |
| Ulnar deviation of left hand | 49.8 | 10.9 |
| Total deviation of left hand | 81.6 | 15.0 |
| Radial deviation of right hand | 30.1 | 7.9 |
| Ulnar deviation of right hand | 49.9 | 10.4 |
| Total deviation of right hand | 80.0 | 14.9 |

15a) Athletes (N=83)

|  | Mean | St Deviation |
| --- | --- | --- |
| Radial deviation of left hand | 28.7 | 6.0 |
| Ulnar deviation of left hand | 52.1 | 6.1 |
| Total deviation of left hand | 80.8 | 9.8 |
| Radial deviation of right hand | 31.7 | 7.4 |
| Ulnar deviation of right hand | 51.6 | 10.9 |
| Total deviation of right hand | 83.3 | 11.8 |

15b) Fracture (N=48)

|  | Mean | St Deviation |
| --- | --- | --- |
| Radial deviation of left hand | 29.3 | 6.7 |
| Ulnar deviation of left hand | 44,2 | 11.6 |
| Total deviation of left hand | 73.5 | 15.2 |
| Radial deviation of right hand | 27.0 | 8.7 |
| Ulnar deviation of right hand | 44.8 | 10.6 |
| Total deviation of right hand | 71.8 | 17.1 |

15c) Inflammation (N=53)

|  | Mean | St Deviation |
| --- | --- | --- |
| Radial deviation of left hand | 41.0 | 20.2 |
| Ulnar deviation of left hand | 45.7 | 22.5 |
| Total deviation of left hand | 86.7 | 41.9 |
| Radial deviation of right hand | 38.0 | 8.9 |
| Ulnar deviation of right hand | 48.7 | 21.8 |
| Total deviation of right hand | 86.7 | 23.9 |

15d) Manual workers (N=38)

|  | Mean | St Deviation |
| --- | --- | --- |
| Radial deviation of left hand | 29.0 | 8.3 |
| Ulnar deviation of left hand | 48.4 | 10.8 |
| Total deviation of left hand | 77.4 | 14.3 |
| Radial deviation of right hand | 28.4 | 8.2 |
| Ulnar deviation of right hand | 47.0 | 9.7 |
| Total deviation of right hand | 75.4 | 14.8 |

15e) Musicians (N=139)

|  | Mean | St Deviation |
| --- | --- | --- |
| Radial deviation of left hand | 29.1 | 7.3 |
| Ulnar deviation of left hand | 46.9 | 109 |
| Total deviation of left hand | 76.0 | 138 |
| Radial deviation of right hand | 28.3 | 8.5 |
| Ulnar deviation of right hand | 48.3 | 9.9 |
| Total deviation of right hand | 76.6 | 12.8 |

5f) Men (N=157)

|  | Mean | St Deviation |
| --- | --- | --- |
| Radial deviation of left hand | 28.6 | 9.3 |
| Ulnar deviation of left hand | 44.6 | 11.7 |
| Total deviation of left hand | 73.2 | 17.7 |
| Radial deviation of right hand | 27.3 | 7.4 |
| Ulnar deviation of right hand | 45.5 | 11.2 |
| Total deviation of right hand | 72.8 | 16.2 |

5g) Women (N=143)

|  | Mean | St Deviation |
| --- | --- | --- |
| Radial deviation of left hand | 29.0 | 8.5 |
| Ulnar deviation of left hand | 45.8 | 11.3 |
| Total deviation of left hand | 74.8 | 16.0 |
| Radial deviation of right hand | 28.0 | 8.0 |
| Ulnar deviation of right hand | 46.7 | 10.7 |
| Total deviation of right hand | 74.7 | 14.9 |

5h) Right- handed (N=255)

|  | Mean | St Deviation |
| --- | --- | --- |
| Radial deviation of left hand | 27.5 | 3.8 |
| Ulnar deviation of left hand | 46.1 | 10.1 |
| Total deviation of left hand | 73.6 | 12.0 |
| Radial deviation of right hand | 26.0 | 6.5 |
| Ulnar deviation of right hand | 50.5 | 8.5 |
| Total deviation of right hand | 76.5 | 9.7 |

5i) Left-handed (N=45)
